# A randomized trial comparing the pharmacology of magnesium sulfate when used to treat severe preeclampsia with serial intravenous boluses versus a continuous intravenous infusion

**DOI:** 10.1186/s12884-018-1919-6

**Published:** 2018-07-06

**Authors:** Thomas Easterling, Mary Hebert, Hillary Bracken, Emad Darwish, Mohamed Cherine Ramadan, Salwa Shaarawy, Dyanna Charles, Tamer Abdel-Aziz, Ahmed Shokry Nasr, Sherif Mohamed Safwal, Beverly Winikoff

**Affiliations:** 1 Department of Obstetrics and Gynecology, University of Washington, 1959 Pacific Street NE, Box 356460, Seattle, WA 98195 USA; 2 School of Pharmacy, University of Washington, Seattle, WA USA; 3 Gynuity Health Projects, New York, NY USA; 4 Shatby Maternity Hospital, Alexandria University, Alexandria, Egypt; 5 El Galaa Teaching Hospital, Cairo, Egypt

**Keywords:** Preeclampsia, Magnesium sulfate, Pharmacokinetics

## Abstract

**Background:**

Magnesium sulfate is the preferred pharmacological intervention for the prevention and treatment of eclamptic seizures in pregnancy. Pain associated with intramuscular injections and the need for an electronic infusion pump for use intravenously represent significant barriers to broader utilization. We hypothesize that an alternative regimen based on serial intravenous (IV) boluses can produce serum concentrations comparable to those produced by a continuous infusion.

**Methods:**

An open-label randomized trial was performed at two hospitals in Egypt. Women with severe preeclampsia were eligible and enrolled between January 2015 and February 2016. Two hundred subjects were randomized by random numbers generated centrally in distinct blocks and stratified by study site. They were assigned to a continuous infusion arm, (4 g loading dose with 1 g/hr. continuous infusion) or a serial IV bolus arm, (6 g loading dose with 2 g bolus every 2 h using a Springfusor® pump). Sparsely sampled magnesium serum concentrations were collected, nonlinear mixed effect modeling was conducted and Monte Carlo simulations were used to generate 200 simulated subjects in each treatment arm. The simulated populations were used to determine area under the concentration-time curve (AUC) as a measure of total drug exposure and compared.

**Results:**

Simulated area under the magnesium serum concentration-time curve was significantly higher in the serial IV bolus arm than in the continuous infusion arm (1107 ± 461 mmol•min /L vs. 1010 ± 398 mmol•min /L, (*P* = 0.02)). Four percent of women in the serial bolus arm considered the treatment unacceptable or very unacceptable compared to 2% in the continuous infusion arm, (*P* = 0.68).

**Conclusions:**

Serial IV boluses achieve serum magnesium concentrations statistically significantly higher but clinically comparable to those achieved with a continuous infusion and offer a third option for the administration of MgSO_4_ to women with preeclampsia that may reduce barriers to utilization.

**Trial registration:**

Trial no. NCT02091401, March 17, 2014.

## Background

Magnesium sulfate (MgSO_4_), the preferred pharmacological intervention for treatment of eclamptic seizures, has been shown to have greater efficacy and fewer complications than treatment with diazepam or phenytoin. [1] MgSO_4_ is also superior to placebo and nimodipine for prevention of eclampsia in women diagnosed with preeclampsia. [2, 3] In the MAGPIE Trial [2] the incidence of eclampsia was reduced in women with preeclampsia: RR 0.42; 95% CI (0.29–0.60). A strong trend towards reduction in maternal mortality was also observed: RR 0.55; CI (0.26–1.14). Study subjects were treated with either an intramuscular (IM) or an intravenous (IV) regimen. The route of administration was not demonstrated to affect drug effectiveness.

Despite established efficacy, utilization of MgSO_4_ remains limited. Barriers to use include: the availability of MgSO_4_, persistent provider attitudes regarding safety and efficacy, lack of availability of infusion pumps for safe intravenous (IV) administration, and pain associated with intramuscular (IM) administration. In a randomized trial comparing IM vs. IV administration, we found that 45% of women receiving IM MgSO_4_ considered pain associated with injections to be unacceptable or very unacceptable compared to only 2% who were treated intravenously. [4] Pain associated with IM MgSO_4_ represents a considerable barrier to use and is potentially addressable.

We hypothesized that by administering serial intravenous (IV) boluses of MgSO_4_ after the loading dose instead of a continuous infusion or IM injections, we could achieve comparable serum magnesium concentrations. The loading dose and serial IV boluses could be given as a manual push by a health care provider or with a simple mechanical flow controlled intravenous pump (Springfusor®). The use of serial IV boluses would eliminate the possibility of an inadvertent overdose from a free running MgSO_4_ IV infusion and circumvent the need for expensive electronic infusion pumps in resource poor environments. If administered with a Springfusor® pump, a single set of flow control tubing would be required rather than one for IV bolus and a second for continuous infusion. This would reduce cost as well as eliminate the potential for inadvertent use of the incorrect flow control tubing. IV administration would significantly reduce maternal pain as a barrier to care.

Can serial IV boluses maintain sufficient serum magnesium concentrations? Abadde et al. tested administration of MgSO_4_ by serial IV bolus compared to continuous infusion in a randomized trial of 30 women with severe preeclampsia or eclampsia. [5] They found that serum magnesium AUC in the serial IV bolus arm was significantly lower than the AUC in the continuous infusion arm and concluded that use of the alternative serial IV bolus regimen might result in an increased risk for seizures and should not be used.

Using population PK modeling and simulations from data in our Springfusor trial, we found that maternal serum magnesium levels were lower in the IV arm compared to the IM arm. [6] After modeling the data, we suggested that use of a 6 g loading dose instead of a 4 g loading dose in the IV regimen would result in serum concentrations comparable to those achieve with the IM regimen. We subsequently evaluated the effect of a 6 g loading dose with a regimen using serial IV boluses through simulations and found that the expected serum concentrations would be comparable to concentrations from the IM and IV arms of the Springfusor trial. In the present study, we tested the use of a 6 g loading dose followed by 2 g IV boluses every 2 h in a randomized trial as compared to continuous infusion.

## Methods

An open-label randomized trial was performed comparing two regimens of administering MgS0_4_ to women with severe preeclampsia who were deemed likely to benefit by the reduction in risk for eclamptic seizures, (trial no. NCT02091401). The trial compared maternal serum magnesium concentrations achieved by each regimen through population pharmacokinetic analysis. The study was conducted at two tertiary-care hospitals in Egypt: El Galaa Teaching Hospital in Cairo and Shatby Maternity Hospital in Alexandria. In El Galaa Hospital 16,000 births are delivered per year. The eclampsia rate is approximately 0.5%. In Shatby Hospital approximately 16,000 births are delivered per year, and the rate of preeclampsia is 6.1%. The study was approved by Ethics Committee of the Faculty of Medicine—Alexandria University and the El Galaa Maternity Teaching Hospital Ethical Committee. Written consent was obtained from each study participant.

To be eligible, women were required to have a systolic blood pressure ≥ 160 mmHg or a diastolic blood pressure ≥ 110 mmHg at two times over 30 min and ≥ 1+ proteinuria. (A single higher pressure could be accepted if the clinical team felt that MgSO_4_ should be initiated without waiting for 30 min.) Eligible women were pregnant or ≤ 24 h postpartum and deemed by the admitting physician to benefit from treatment with MgSO_4_. Women agreed to comply with study procedures. Women were excluded if they had experienced an eclamptic seizure, had received MgSO_4_ within 24 h of study enrollment, or had a serum creatinine > 106 μmol/L (1.2 mg/dL) at the time of enrollment. Subjects gave written informed consent. After informed consent, a sequentially numbered, sealed, opaque envelope containing the participant’s group assignment was opened by research staff. The envelopes were generated by Gynuity Health Projects staff using a randomisation code based on a computerised pseudo-random number generator. Randomisation was stratified by center.

As a population PK study, the sample size (*n* = 100 per group) and samples per subject (*n* = 7) were chosen to rigorously characterize the distribution and elimination of magnesium under both conditions. From this characterization, population PK modeling could then be performed. A large sample was chosen due to anticipated uncertainties regarding variability in the study population. No midterm analysis was conducted for assessment of safety.

In the serial IV bolus arm, magnesium was administered as 50% magnesium sulfate heptahydrate (MgSO_4_•7H2O) [Hospira]. In the continuous infusion arm, 10% magnesium sulfate heptahydrate [Egyptian International Pharmaceutical Industries Company]was used, the community standard. Women were randomized to receive one of two regimens. The CONTINUOUS INFUSION ARM was the community standard at each hospital. A 4 g intravenous loading dose of 10% MgSO_4_ was administered manually over approximately 20 min as done in the MAGPIE Trial. [2] At the conclusion of the loading dose a continuous infusion of MgSO_4_ at 1 g/hour by mini-drip was started (the community standard). In the SERIAL IV BOLUS ARM, 50% MgSO_4_ was administered by Springfusor® spring-loaded pump, (GoMedical, Subiaco, Australia), through a flow control tubing designed to deliver 10 mL of saline over 5 min and calibrated to deliver the viscous 50% MgSO_4_ solution to study requirements. A 6 g (12 mL) intravenous loading dose was administered over approximately 30 min. Subsequently, a 2 g (4 mL) IV bolus was administered over 10 min every 2 h. Clinical care was managed by local standards. The loading dose and subsequent IV boluses were delivered through a side port of an IV infusion running at 100-150 ml per hour to achieve adequate dilution of the 50% solution. The delivery of 50% solution was only 4 ml per minute.

Maternal serum samples were collected over a 12-h infusion period.

Each subject had a baseline and 6 strategically timed blood samples drawn during the study period for measurement of magnesium concentrations. The samples were distributed to be informative for a population pharmacokinetic (PK) analysis. Sample collection times are in relation to the end of the loading dose. Subjects in the continuous infusion arm were assigned to one of three groups for sample collection times. Continuous Infusion Group-1 (*n* = 33) collected samples at time Baseline, 20, 80, 160, 280, 500 and 740 min. Continuous Infusion Group-2 (n = 33) collected samples at time Baseline, 60, 120, 240, 420, 580 and 740 min. Continuous Infusion Group-3 (*n* = 34) collected samples at time Baseline, 40, 100, 200, 340, 660 and 740 min. Subjects in the serial IV bolus arm were also assigned to one of three groups for sample collection times. Serial Bolus Group-1 (n = 33) collected samples at time Baseline, 20, 100, 200, 380, 660 and 750 min. Serial Bolus Group-2 (n = 33) collected samples at time Baseline, 40, 140, 300, 400, 500 and 600 min. Serial Bolus Group-3 (n = 34) collected samples at time Baseline, 60, 160, 260, 340, 440 and 620 min. Actual drug administration and sampling times were used in the analysis. A serum creatinine level was drawn at baseline and at the conclusion of the study period. At Shatby hospital, magnesium levels were measured in the hospital laboratory with a Cobass 501 system using a colorimetric endpoint method (*Roche Diagnostics*). At El Galaa Hospital, magnesium levels were measured in the hospital laboratory with BT-3500 autoanalyzer using colorometric endpoint method, (*Biotecnia Instruments SpA*).

At the conclusion of treatment with MgSO_4_, subjects were interviewed and asked to grade side effects as mild, moderate or severe. They were also asked to rate the overall acceptability of side effects as “very acceptable,” “acceptable,” “neutral,” “unacceptable” or “very unacceptable”. (This approach, while used extensively, has not been validated for reproducibility in our hands).

Serum magnesium concentrations were analyzed with nonlinear mixed effect model using NONMEM (version 7.3.0). [7] One and two-compartmental models with first order elimination were evaluated for goodness-of-fit diagnostics. Maximum likelihood population parameter estimates were determined for clearance (CL), volume of distribution (V) and baseline endogenous steady-state magnesium concentration (BL). The administered magnesium was modeled as additive to BL. Combined data as well as continuous infusion data and short infusion data were modeled for goodness of fit. Combined data was used for the final model.

After determination of the base model, maternal age, actual body weight, ideal body weight, height, gestational age, serum creatinine at the start of the PK study, serum creatinine at the end of the PK study, creatinine clearance at the start of the PK study, creatinine clearance at the end of the PK study and number of fetuses were tested as linear, additive, exponential, proportional and power model covariates as appropriate for potential inclusion in the final model. Creatinine clearance was determined using the Cockcroft and Gault equation. [8] Weight adjustments were normalized for the mean body weight in the study (90.54 kg). The final model inclusion of a covariate was based on statistical significance with a decrease in objective function of at least > 3.84 (*P* < 0.05).

Following determination of the final model, Monte Carlo simulation was used to generate a continuous infusion population with 200 subjects and a serial IV bolus population with 200 subjects receiving the dosing for the original study design as described above. The simulations utilized the final population model parameter estimates of fixed effects as well as inter-individual and residual random variability. Body weight was varied within a range of ±30% of the mean body weight. The 12 h and 20 min area under the concentration-time curve was estimated for each group using linear trapezoidal rule.

The study was funded by Merck for Mothers who had no role in study design, data collection, data analysis, data interpretation, or writing of the report. The corresponding author had full access to all data in the study and had final responsibility for the decision to submit for publication.

## Results

**Fig. 1:**
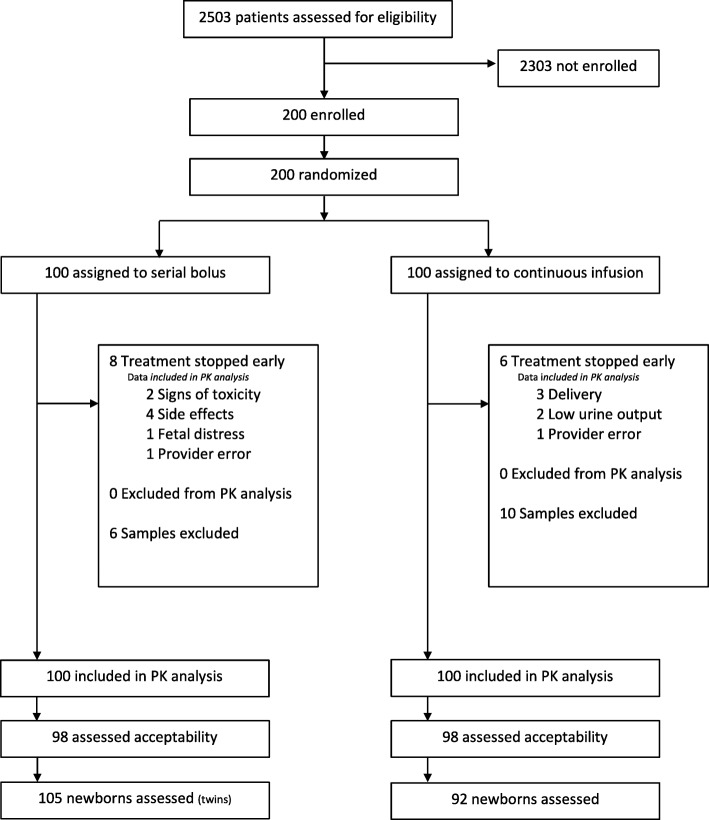
Consort Diagram

Two hundred women were enrolled in the study, 100 from El Galaa Teaching Hospital in Cairo and 100 from Shatby Maternity Hospital in Alexandria between January 2015–February 2016. The Consort Diagram is displayed in Fig. 1. Table 1 describes maternal characteristics. Only the incidence of primigravidity appeared different between groups. Table 2 describes maternal outcomes that were similar between groups. No adverse maternal outcomes such as eclampsia, maternal death, or the need for transfusion were experienced. In the serial IV bolus arm, 8 women stopped treatment early due to side effects (*n* = 4), signs of toxicity (*n* = 2), fetal distress (*n* = 1) or provider error (*n* = 1). In cases where treatment was stopped early due to signs of toxicity (bradycardia, decreased respiratory rate, or absent reflexes), calcium gluconate was administered, women recovered fully and no ICU admission was required. In the continuous infusion arm, 6 women stopped treatment early due to delivery (*n* = 3), low urinary output (*n* = 2) or provider request (*n* = 1).

**Table 1:** Maternal Characteristics

			Serial IV Bolus	Continuous Infusion
			(*n* = 100)	(*n* = 100)
Maternal age (years) mean ± SD			29 ± 6	29 ± 6
Weight (kg) mean ± SD			91.4 ± 17.2	89.6 ± 14.8
Gestational Age (weeks) mean ± SD			35.7 ± 2.8	35.2 ± 3.3
Primigravid - N (%)			46 (46%)	24 (24%)
Multiple Pregnancy - N (%)			5 (5%)	4 (4%)
Postpartum enrollment- N (%)			0 (0%)	2 (2%)
Systolic BP (mmHg) mean ± SD			167 ± 10.9	165 ± 7.7
Diastolic BP (mmHg) mean ± SD			113 ± 7.7	113 ± 5.9
Proteinuria - N (%)	< 1+		0	0
	1+		10 (10%)	17 (17%)
	≥2+		90 (90%)	83 (83%)
Serum creatinine				
Initial	(μmol/L)	mean ± SD	66.3 ± 15.9	66.3 ± 15.9
		range	26.5–106	26.5–106
	(mg/dL)	mean ± SD	0.75 ± 0.18	0.75 ± 0.18
		range	0.3–1.2	0.3–1.2
Final	(μmol/L)	mean ± SD	73.4 ± 15.9	74.3 ± 16.8
		range	44.2–133	26.5–141
	(mg/dL)	mean ± SD	0.83 ± 0.18	0.84 ± 0.19
		range	0.50–1.5	0.30–1.6

**Table 2:** Maternal Outcomes

		Serial IV Bolus	Continuous Infusion
		(*n* = 100)	(*n* = 100)
Discharged prior to delivery - N (%)		0	6 (6%)
Missing Data		1	3
		(*n* = 99)	(*n* = 91)
Labor induced - N (%)		17 (17.2%)	10 (11%)
Mode of delivery	Vaginal- N (%)	22 (22.2%)	15 (16.5%)
	Forceps - N (%)	1 (1.0%)	3 (3.2)
	Cesarean - N (%)	76 (76.8%)	73 (80.2%)
Postpartum Hemorrhage - N (%)		1 (1.0%)	0
Blood products after trial entry - N (%)		0	0
Seizures - N (%)		0	0
Maternal Deaths - N (%)		0	0

Table 3 describes neonatal outcomes that were similar between groups. Complications were largely those related to preterm birth.

**Table 3:** Neonatal Outcomes

	Serial IV Bolus	Continuous Infusion
Mothers enrolled	*n* = 100	*n* = 100
Enrolled postpartum	0	2
Discharged prior to delivery	0	6
Twin Infants	6	2
Missing data	1	2
Newborns evaluated	*n* = 105	*n* = 92
Live Birth- N (%)	105 (100%)	91 (98.9%)
Stillbirth- N (%)	0	1 (1.1%)
Intubated at delivery - N (%)	6 (5.7%)	6 (6.5%)
Mechanical Ventilation - N (%)	3 (2.8%)	6 (6.5%)
Bradycardia < 110/min - N (%)	2 (1.9%)	4 (4.3%)
Special Care Nursery - N (%)	25 (23.8%)	26 (28.2%)
Neonatal Deaths - N (%)	2 (1.9%)	3 (3.2%)
Prematurity	0	1
Unknown	2	2

From the 200 study subjects, 1347 magnesium concentrations were obtained. Of these, 16 were excluded from analysis for one of two reasons: 1) the concentrations appeared to be contaminated by the magnesium infusion or 2) the concentration at time zero was higher than all other concentrations during the study suggesting the sample was mislabeled or contaminated by the infusion.

**Fig. 2:**
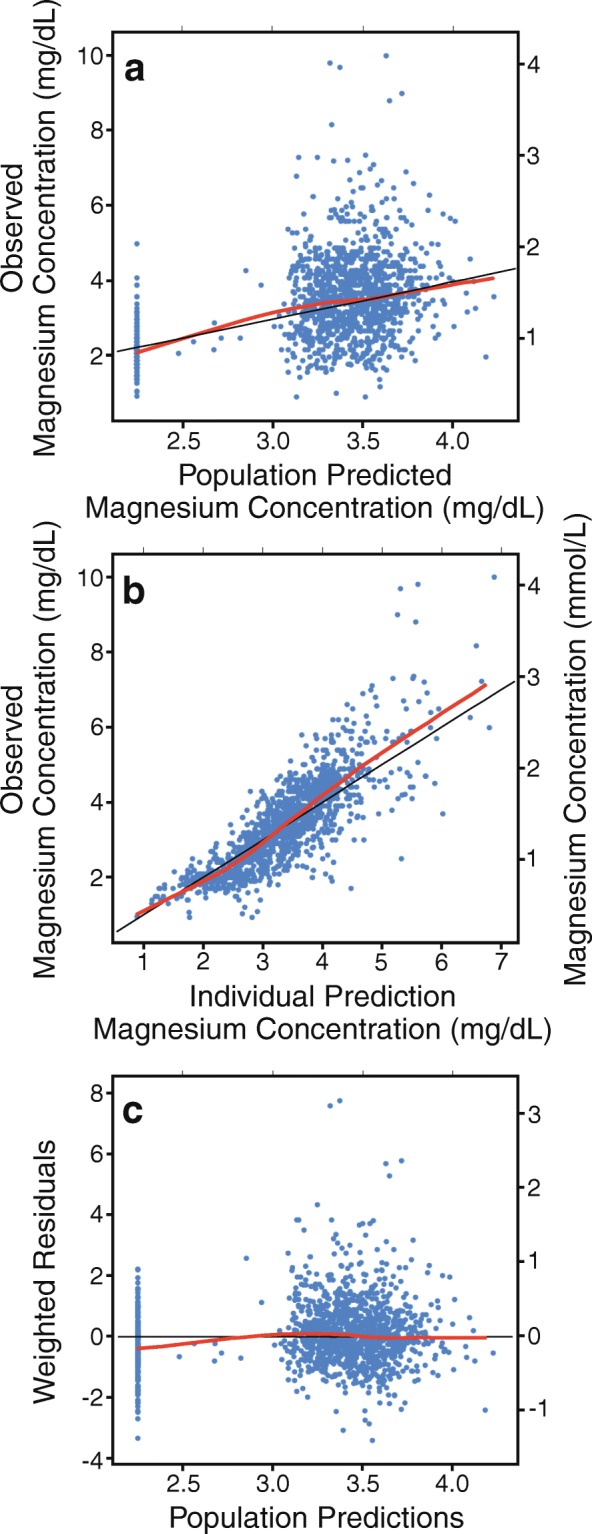
Observed vs. predicted magnesium concentrations in final model. The RED line represents the regression results. The BLACK line is the line of unity where X = Y

A one-compartment linear elimination model provided the best fit of the data. Using weight (exponential) as a covariate for volume and serum creatinine at the start of the study (linear) as a covariate for clearance provided the greatest decrease in the objective function and were utilized in the final model. Fig. 2A depicts a scatterplot of observed magnesium concentrations versus model-population predicted magnesium concentrations for the final model. Fig. 2B depicts a scatterplot of the observed magnesium concentrations versus model-individually predicted magnesium concentrations. Fig. 2C depicts weighted residuals versus model predicted concentrations demonstrating a fairly symmetric distribution. Comparing the diagnostic plots for base and final models demonstrate that the inclusion of weight as a covariate for volume and serum creatinine as a covariate for clearance substantially improves the prediction of magnesium concentrations. However, unexplained clinical variability in magnesium concentrations was still apparent (data not shown).

Pharmacokinetic parameters estimated from the continuous infusion subjects alone and the serial IV bolus subjects alone were comparable to parameters estimated using all data combined (data not shown), therefore all data combined was used in the final model. Table 4 reports the estimated average clearance, volume of distribution and baseline magnesium concentration from the population analysis. Also reported are the effects of weight on magnesium volume and serum creatinine at the start of the study on magnesium clearance.

**Table 4:** Population pharmacokinetic model parameters – mean ± SE

Clearance (mL/min)		56.3 ± 12.7
Volume of distribution (L)		65.3 ± 13.1
Baseline Mg concentration	(mmol/L)	0.925 ± 0.037
	(mg/dL)	2.25 ± 0.09
Weight effect on volume exponential (θ_1_)		−0.426 ± 0.196
Serum creatinine effect on exponential (θ_1_)		0.553 ± 0.138
Residual variability		0.0387 ± 0.0013

**Fig. 3:**
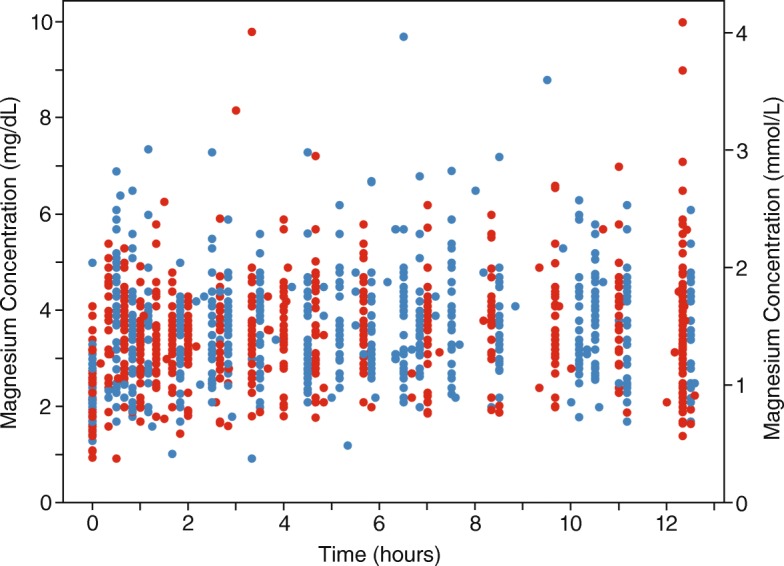
Individual magnesium concentrations over time. A-Continuous Infusion. B-Serial IV Bolus

Figure 3 depicts the actual individual magnesium concentrations plotted vs. time where time zero is at the initiation of the loading dose. Blue dots represent the subjects receiving serial IV boluses and red dots represent the subjects receiving a continuous infusion. (This graph excludes one subject that had a deviation from study protocol that resulted in samples obtained well beyond the end of the study period. Data was included in the PK analysis). The graphs demonstrate a broad overlap in magnesium concentrations in the two treatment arms.

**Fig. 4:**
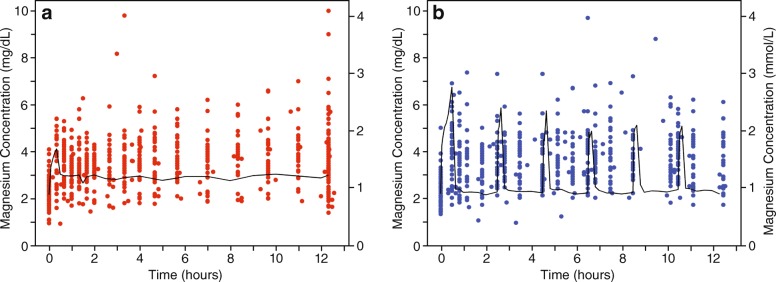
Median simulated magnesium concentrations over time with observed magnesium concentrations. A-Continuous Infusion. B-Serial IV Bolus

Figure 4 demonstrates the appropriateness of the model in describing the data. Figure 4A depicts the median simulated magnesium concentration-time profile for the continuous infusion arm (black line) and the observed magnesium concentrations (red dots). Fig. 4B depicts the median simulated magnesium concentration-time profile for the serial IV bolus arm (black line) and the observed magnesium concentrations (blue dots).

**Fig. 5:**
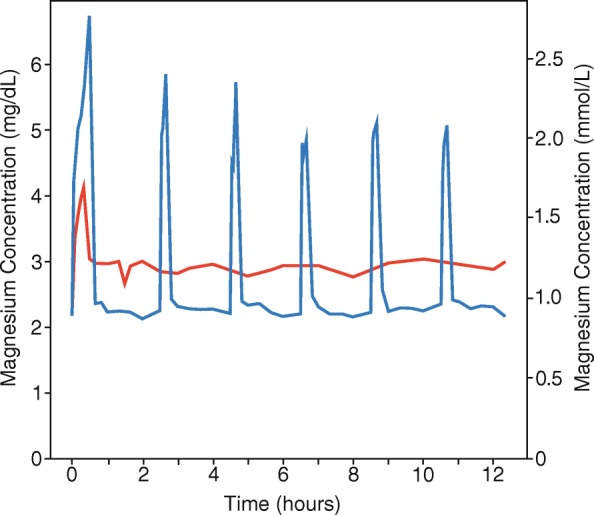
Median simulated magnesium concentrations over time. Red-Continuous Infusion. Blue-Serial IV Bolus

Figure 5 depicts the median magnesium concentration time-curves for the continuous infusion simulated population (red line, *n* = 200) and the serial IV bolus simulated population (blue line, n = 200). The average (± SD) simulated 12 h and 20 min magnesium AUC was 1010 ± 398 mmol•min /L, (2458 ± 969 mg•min/dL), for the continuous infusion arm and 1107 ± 461 mmol•min /L, (2694 ± 1123 mg•min/dL), for the serial IV bolus arm (*P* = 0.02). Mean magnesium serum concentration was derived by dividing the AUC by the total time. Mean concentration in the serial IV bolus arm was 1.49 mmol/L (3.62 mg/dL) compared to 1.36 mmol/L (3.32 mg/dL) in the continuous infusion arm.

Table 5 describes maternal side effects and acceptability of treatment associated with each treatment arm. Women receiving serial IV boluses reported more mild to moderate pain, largely at the infusion site during boluses, (*P* < 0.05). There was also a trend towards more flushing in the serial IV bolus group. Only a small number of subjects in each group rated the treatment as unacceptable or very unacceptable.

**Table 5:** Side Effects

	Serial IV Bolus	Continuous Infusion
	*n* = 98	*n* = 98
Nausea- N (%)	10 (10.2%)	8 (8.2%)
Mild	6	6
Moderate	3	2
Severe	1	0
Vomiting – N (%)	5 (5.1%)	2 (2.0%)
Mild	1	0
Moderate	3	2
Severe	1	0
Flushing - N (%)	18 (18.4%)	9 (9.2%)
Mild	16	9
Moderate	0	0
Severe	2	0
Drowsiness - N (%)	15 (15.3%)	13 (13.2%)
Mild	11	7
Moderate	3	6
Severe	1	0
Pain - N (%)	28 (28.6%)	9 (9.2%) (P < 0.05)
Mild	20	9
Moderate	7	0
Severe	1	0
Acceptable/Very acceptable	88 (89.8%)	94 (95.9%)
Neutral	6 (6.1%)	2 (2.0%)
Unacceptable/Very unacceptable	4 (4.0%)	2 (2.0%)

## Discussion

We found that magnesium sulfate administered by serial IV boluses achieved serum magnesium concentrations significantly higher but clinically equivalent to those achieved with a continuous infusion offering a third option for treatment of women with preeclampsia. Magnesium sulfate is the preferred treatment of eclamptic seizures and for the prevention of seizures in women with preeclampsia. Our goal was to reduce barriers to treatment with MgSO_4_ by developing an alternative regimen that would avoid the pain and lack of acceptability of intramuscular injections and the need for an electronic pump to safely regulate an intravenous infusion. A clinical trial based on the incidence of seizures was not deemed feasible. To examine a potential 50% increase in the incidence of eclampsia from 0.8% reported in the MgSO_4_ arm of the MAGPIE Trial to 1.2% would require a sample size of more than 9000 subjects in each arm. We therefore chose to test pharmacological equivalence by comparing the serum AUC achieved by each regimen.

We found that a one-compartment linear elimination model provided the best fit of the data, similar to prior studies. [6, 9] Body weight and serum creatinine improved model fit as previously described. [6] Our estimated clearance, 56.3 mL/min, was similar to those previously reported by Brookfield et al., (66.3 mL/min), Salinger et al., (80.1 mL/min), and Chuan et al., (71.3 mL/min) [6, 9, 10]. However, our volume of distribution, (65.5 L), and body weight, (90.5 kg), were higher than previously reported by Brookfield et al. (22.5 L; 87.5 kg), Salinger et al. (15.6 L; 57.3 kg), Lu et al. (49 L; 68 kg) and Chuan et al. (32.3 L; 65 kg, pre-pregnancy)[6, 9–11].

The degree of variability described in Fig. 3 demonstrates a fundamental challenge to dosing MgSO_4_ empirically without reference to individual serum magnesium concentrations. Nevertheless, this is the only feasible approach in low resource settings. We have previously reported a similar variability in a cohort of Indian women. [6] This residual variability in the model unexplained by maternal weight or renal function is likely due to differences in volume of distribution associated with differences in extracellular fluid including plasma volume and edema. Any difference in magnesium exposure associated with different regimens must be interpreted in context of the background variability. The observed variability poses an inherent challenge in achieving appropriate dosing – neither too low nor too high – with a uniform dosing strategy.

The total magnesium exposure, as measured by AUC, using simulated populations was higher in the serial IV bolus group, (Fig. 5). Higher peak concentrations associated with serial IV boluses were offset by lower trough concentrations. Although the AUC was significantly higher in the serial IV bolus arm, the mean differences are probably not clinically significant in the context of observed variability. Does the higher AUC in the serial IV bolus arm represent equivalent or superior efficacy despite lower trough concentrations? Given a lack of understanding of the mechanism of seizure prevention associated with magnesium, this question is difficult to answer but worthy of consideration. If the mechanism of action is due to vasomotor relaxation exhibited by patient flushing, serial IV boluses could be more effective. If the site of action is outside the blood stream, the high serum levels during IV boluses would drive a more rapid redistribution into the tissue and might be more effective. Alternatively, if efficacy is dependent on steady state serum concentration, the serial IV bolus regimen might be less effective. As previously discussed, the magnitude of variability between subjects probably overshadows these arguments.

The study was not powered to detect differences in maternal and neonatal outcomes. We did not find any suggestion of worse outcomes for mothers receiving serial IV boluses. Although serial IV boluses resulted in a higher AUC, the magnitude of these differences was small in the context of the variability in serum level. The observed high serum levels were, on inspection, distributed comparably across study groups. MgSO_4_ infusions were stopped early in 8% of women in the serial IV bolus arm compared to 6% in the continuous infusion arm. This compares to a discontinuation rate of 16% in the treatment arm of the MAGPIE Trial. [2] The incidence of neonates requiring mechanical ventilation in the serial IV bolus arm, (2.8%) was lower than that in the continuous infusion arm (6.5%). The incidence of bradycardia in the serial IV bolus arm (1.9%) was lower than that in the continuous infusion arm (4.3%). These results suggest that an increased incidence of adverse outcomes using serial IV boluses is unlikely.

The Springfusor® pump was used to achieve a standardized infusion rate in the serial IV bolus arm. The flow-control tubing effectively prevents an accidental rapid infusion and does not require constant staff attention. Serial IV boluses could be performed manually at the same rate by a health care provider in the absence of a Springfusor® pump but would be subject to variability in infusion rate and would require greater use of provider resources. The serial IV bolus approach could be adapted to the concentration of MgSO_4_ used at a specific site and to the local method used to administer an IV loading dose. Local conditions, balancing the cost and availability of health care staff against the cost and availability of the Springfusor® pump system, would drive local decision making.

Few women in either arm found the treatment unacceptable or very unacceptable though women in the serial IV bolus reported more pain, generally mild to moderate. Pain was at the infusion site. Unacceptable pain or very unacceptable pain (4.0%) was much less than we have reported with use of IM MgSO4 (29.4%). [4] While inclusion of lidocaine with IM MgSO4 may improve acceptability above that seen in our prior trial, it is unlikely to impact pain of the injection itself. There was also a tendency to report more flushing. By decreasing the infusion rate with a slower flow control tubing, we might be able to decrease pain at the infusion site and flushing. This strategy would also decrease peak concentrations associated with each IV bolus without affecting the total magnesium exposure. Increasing infusion rate of the back-up IV will also serve to decrease pain with infusion.

This study was conducted in lower resource settings, the intended site of potential use, which could pose inherent challenges in performing a pharmacokinetic study. Some individual serum samples were excluded due to what appeared to be protocol violations and may have been due to a lapse in appropriate attention to blood draws at the beginning of the protocol. This affected only 1.1% of total samples.

We have demonstrated that serial IV boluses represent a third option for the administration of MgSO_4_ to women with preeclampsia. Serial IV boluses achieve serum magnesium concentrations significantly higher but clinically equivalent to those achieved with a continuous infusion. This strategy avoids barriers to care associated with painful IM injections and the need for an electronic continuous infusion pump, not readily available in low resource settings. It can also be initiated prior to transport of a patient to a higher level of care without the need to treat during transport. The use of a Springfusor® pump, while fundamentally not required, insures the consistency of infusion rate and prevents an overly rapid manual infusion if administered by a provider.

## Conclusions

Serial IV boluses can be used to administer MgSO_4_ to pregnant women at risk for eclampsia. Adoption of this strategy may overcome barriers associated with IM injections and continuous infusions.

## Abbreviations

AUC: area under the curve
BL: baseline
CL: clearance
dL: deciliter
g: gram
hr.: hour
IM: intramuscular
IV: intravenous
MgSO_4_: magnesium sulfate
MgSO4•7H20: magnesium sulfate heptahydrate
min: minute
mL: milliliter
mmHg: millimeters of mercury
mmol: millimole
PK: pharmacokinetic
V: volume of distribution
μmol: micromole
